# Supplementary material for: Staphylococcus aureus β-Toxin Exerts Anti-angiogenic Effects by Inhibiting Re-endothelialization and Neovessel Formation
Source: Front Microbiol. 2022 Feb 3;13:840236. doi: 10.3389/fmicb.2022.840236 (PMC8851161; doi:10.3389/fmicb.2022.840236)
Supplement: Supplementary file 1 [file Data_Sheet_1.PDF]

Supplemental Figure 1

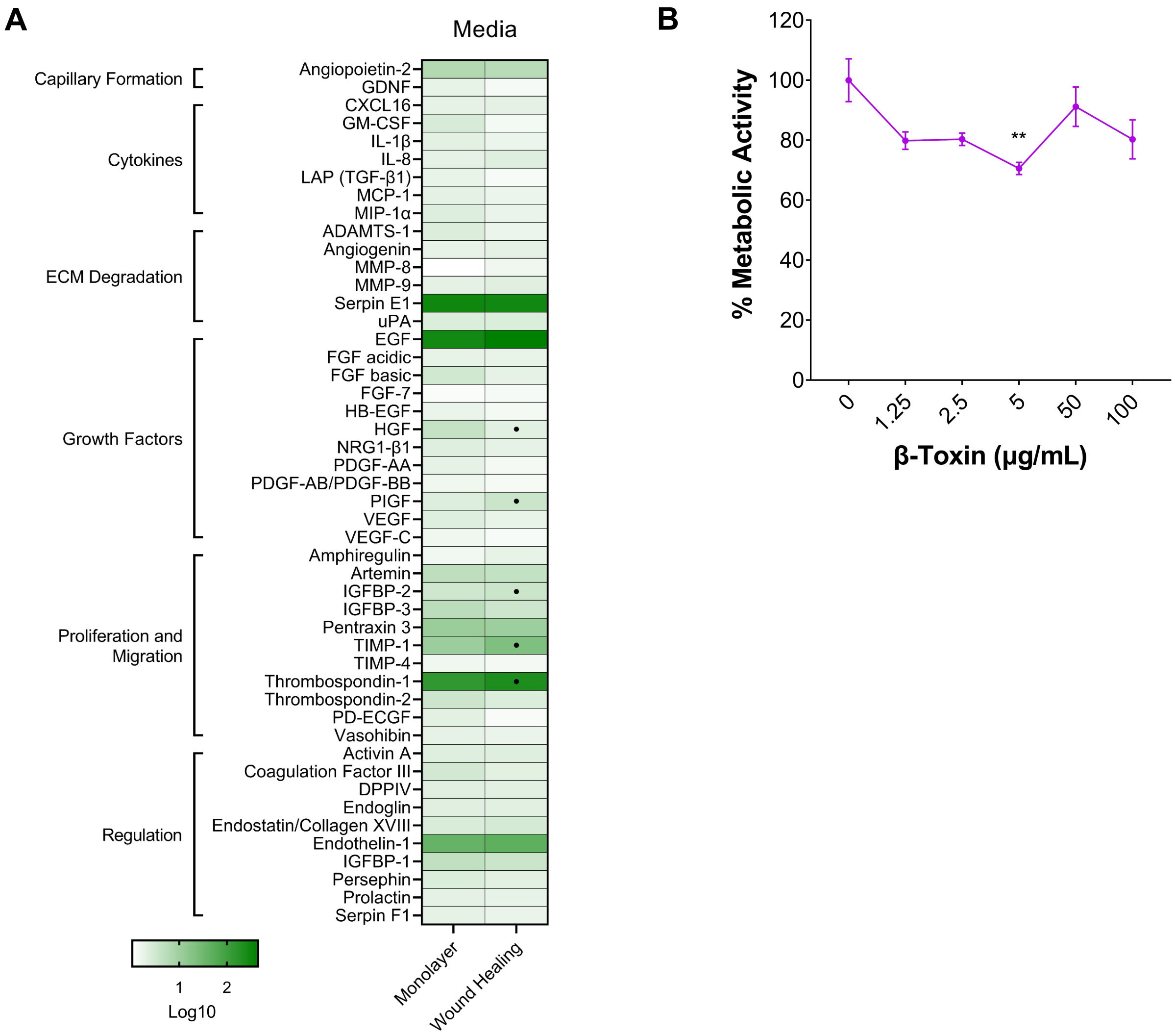

Supplemental Figure 1.

(A) Proteome analysis of untreated iHAECs grown to confluency on 1% gelatin-coated plates. Data is log scale with background threshold removed. • angiogenic-related factors with a 50% increase (>1.5-fold change) or decrease (<0.5-fold change) from media control.

(B) Percent metabolic activity. iHAECs were grown to near confluency on 1% gelatin-coated plates and treated for 24 h with β-toxin (50 µg mL^{-1}). Statistical significance determined by unpaired, two-tailed t-tests compared to untreated (0 µg mL^{-1}).

Supplemental Figure 2

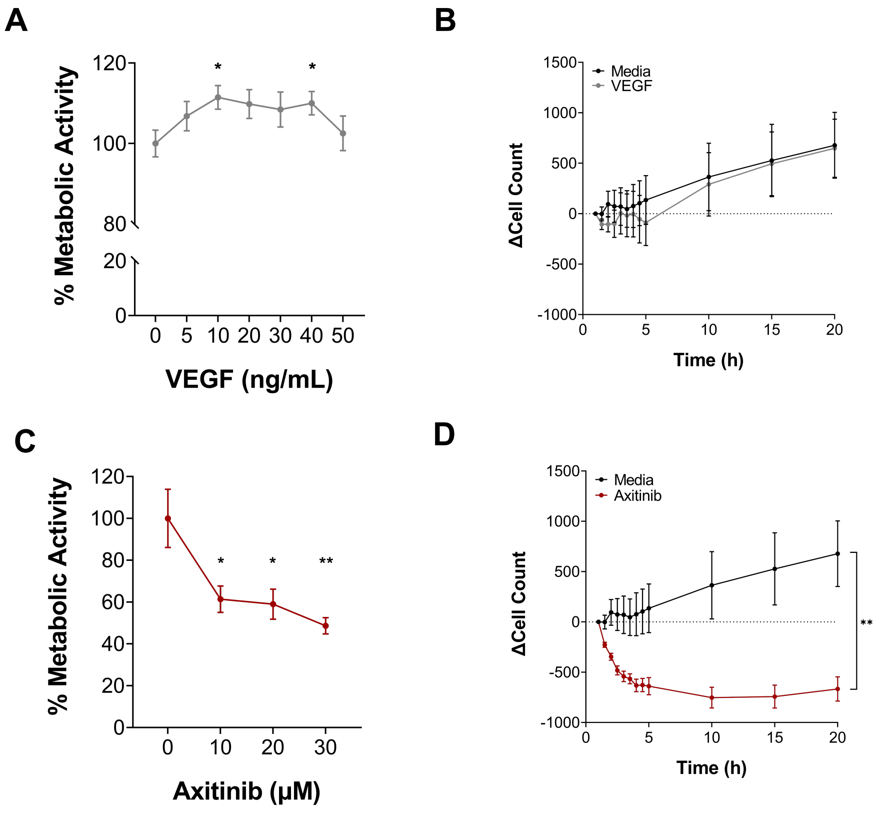

Supplemental Figure 2.

(A, C) Percent metabolic activity. iHAECs were grown to near confluency on 1% gelatin-coated plates and treated for 24 h with (A) VEGF or (C) axitinib. Statistical significance was determined by unpaired, two-tailed t-tests compared to untreated (0 μg mL^{-1}).

(B, D) Cell proliferation of iHAECs seeded at 7,000 cells $well^{-1}$ and treated with (B) VEGF (10 ng mL^{-1}) or (D) axitinib (10 μM) over a 20-h period. Cells counted every 30 min for the first 5 h then every 5 h thereafter. Results represent the change in cell count (mean ± SEM) of three independent experiments conducted in triplicate. Statistical significance determined by unpaired, two-tailed t-test at 20 h.

Supplemental Figure 3

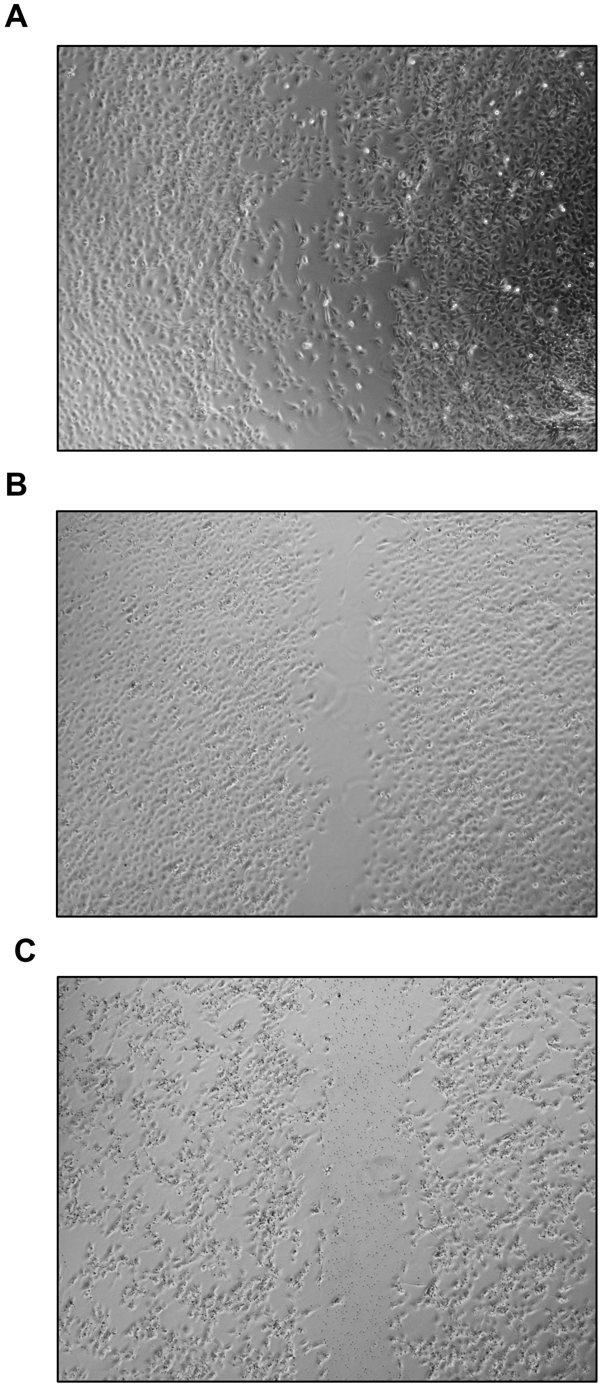

Supplemental Figure 3.

Phase-contrast microscopy of wound healing experiments at 24 h.

(A) Untreated iHAECs.

(B) iHAECs treated with 10 μM axitinib.

(C) iHAECs treated with 30 μM axitinib.

Supplemental Figure 4

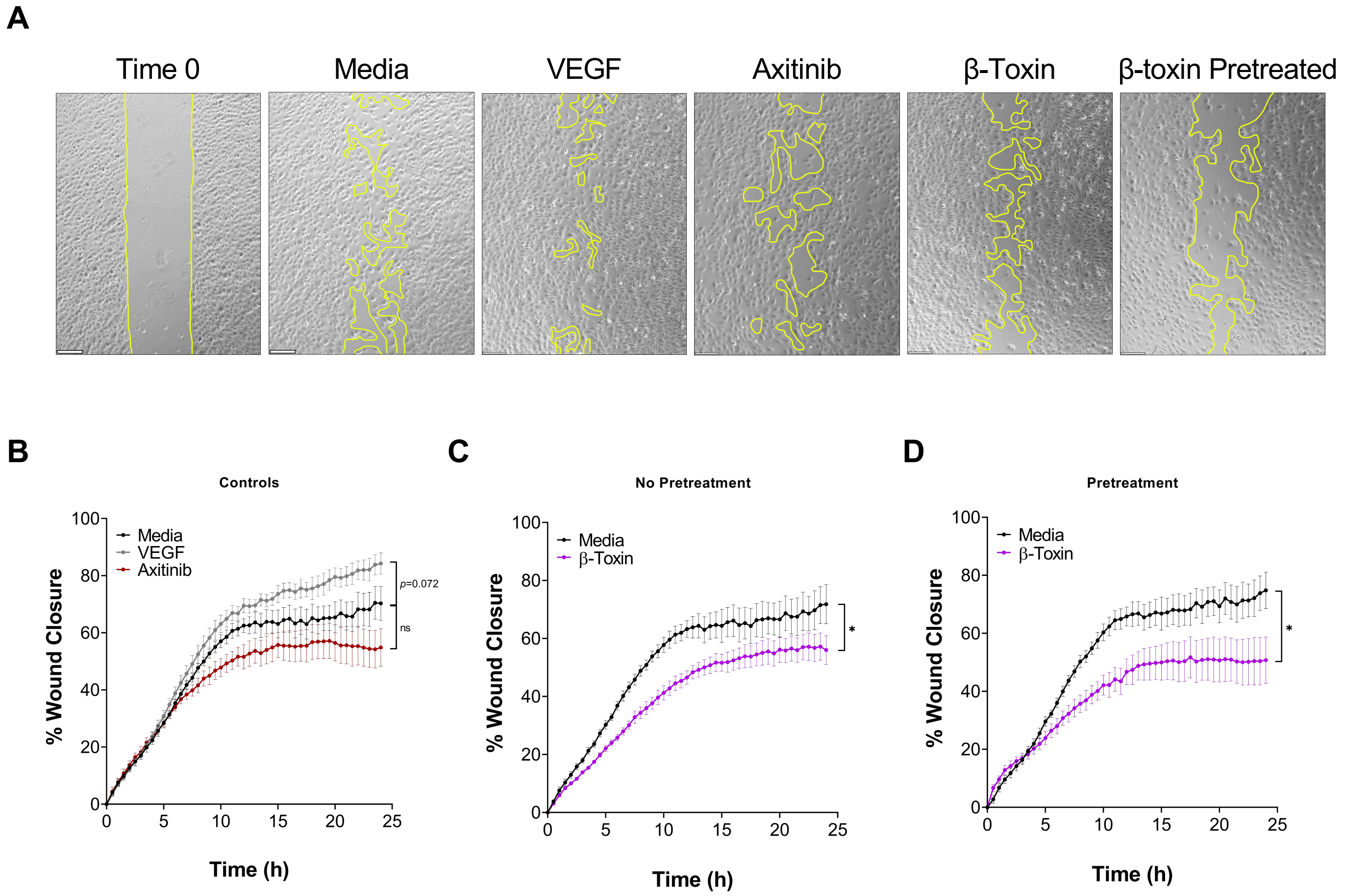

Supplemental Figure 4.

(A) Phase-contrast microscopy Time 0 (representative image) and at 24 h. Images captured every 30 min. Scale bar = 200 µm.

(B) HUVECs treated with VEGF (10 ng mL^{-1}) or axitinib (10 µM) at the start of the experiment.

(C) HUVECs treated with β-toxin (50 µg mL^{-1}) at the start of the experiment.

(D) HUVECs treated overnight with β-toxin (50 µg mL^{-1}) prior to gap formation and thereafter.

(B-D) All results are mean ± SEM of five independent experiments with four replicates each.
* $p < 0.0332$; two-way repeated measures ANOVA.

Supplemental Figure 5

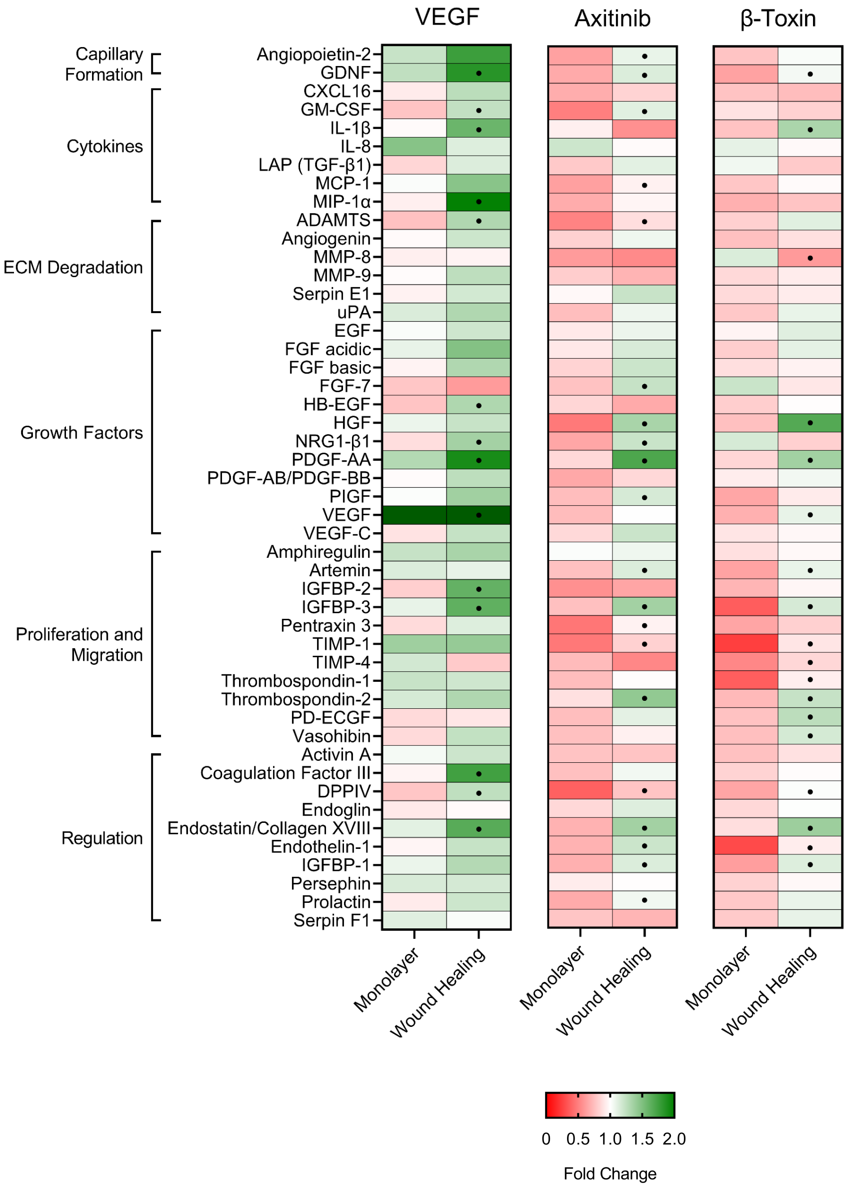

Supplemental Figure 5.

iHAECs treated with VEGF (10 ng mL^{-1}), axitinib (10 μM in wound healing; 30 μM in monolayers), or β-toxin (50 μg mL^{-1}). Data is on a linear scale and is a reproduction of data in Figures 1 and 3. Results are the mean fold change over matched untreated cells. • angiogenic-related factors with a 50% increase (>1.5-fold change) or decrease (<0.5-fold change) from media control.

Supplemental Figure 6

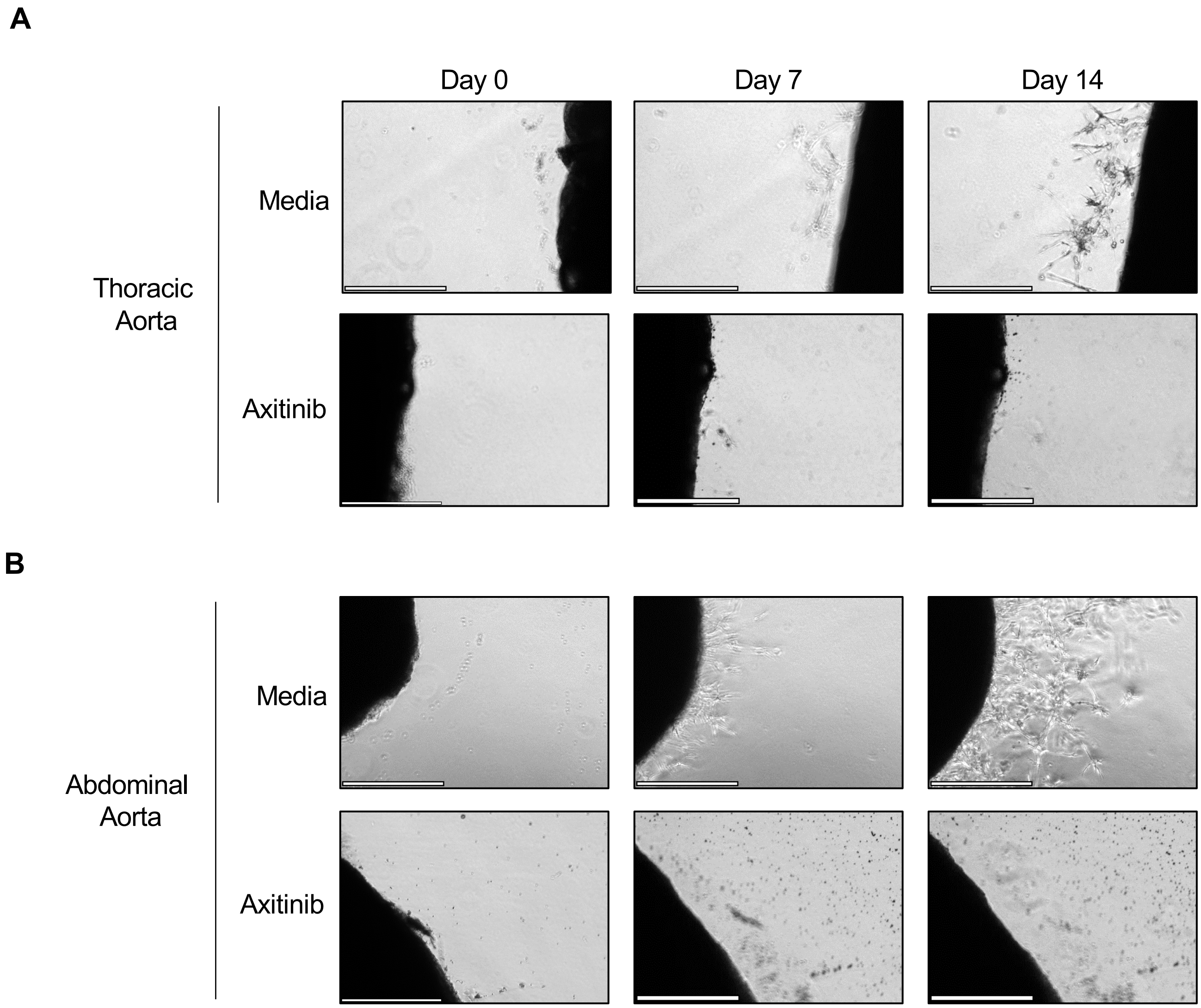

Supplemental Figure 6.

Thoracic and abdominal aortas were collected and sectioned from 2–3 kg New Zealand white rabbits. Rings were cultured on GFR-Matrigel in the presence or absence of axitinib (10 μM). Scale bar = 500 μm.

(A) Phase-contrast microscopy of thoracic aortic rings.

(B) Phase-contrast microscopy of abdominal aortic rings.

Supplemental Figure 7

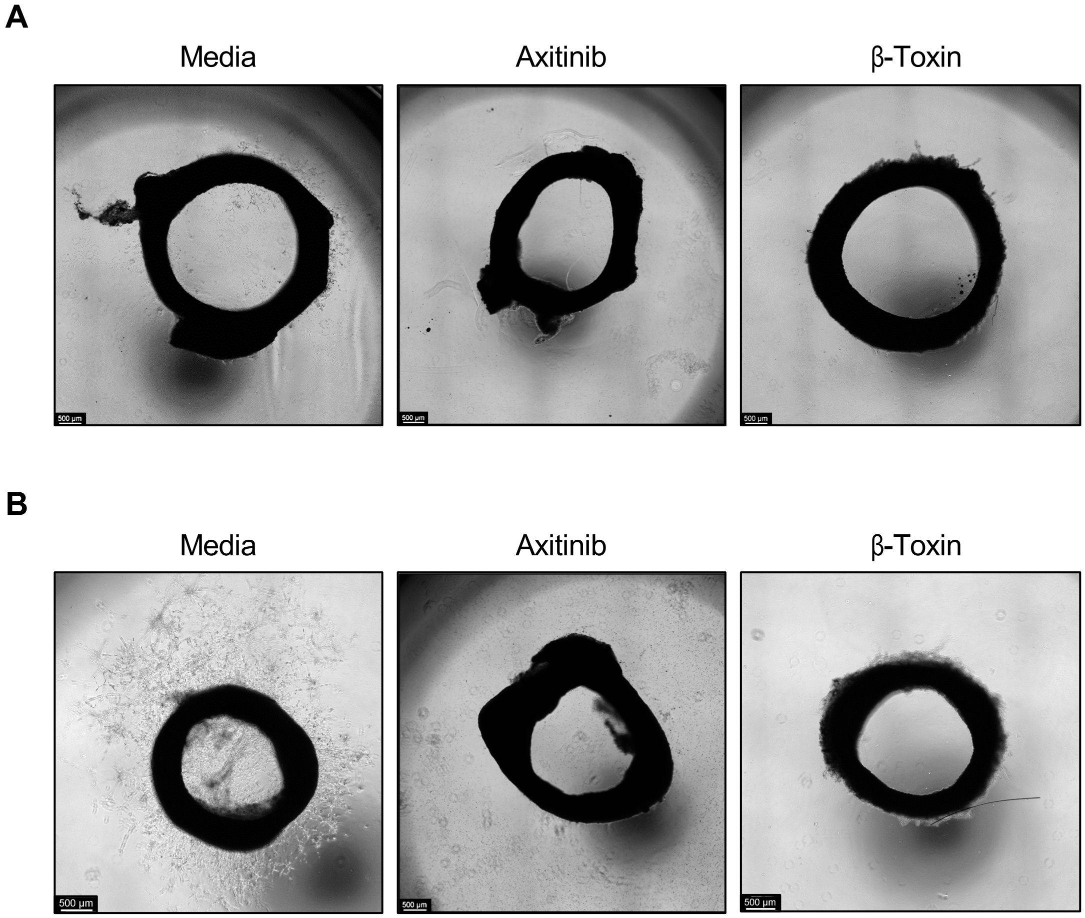

Supplemental Figure 7.

Thoracic and abdominal aortas were collected and sectioned from 2–3 kg New Zealand white rabbits. Rings were cultured on GFR-Matrigel in the presence or absence of axitinib (10 µM). Scale bar = 500 µm.

(A) Phase-contrast microscopy of thoracic aortic rings.

(B) Phase-contrast microscopy of abdominal aortic rings.
